# Neck circumference and waist circumference associated with cardiovascular events in type 2 diabetes (Beijing Community Diabetes Study 23)

**DOI:** 10.1038/s41598-021-88927-9

**Published:** 2021-05-04

**Authors:** Guang-Ran Yang, Ming-Xia Yuan, Gang Wan, Xue-Lian Zhang, Han-Jing Fu, Shen-Yuan Yuan, Liang-Xiang Zhu, Rong-Rong Xie, Jian-Dong Zhang, Yu-Ling Li, Yan-Hua Sun, Qin-Fang Dai, Da-Yong Gao, Xue-Li Cui, Jian-Qin Gao, Zi-Ming Wang, Ying-Jun Chen, Yong-Jin Li, Dong-Ming Hu, Juan Gao, Ying Gao, Jie Miao, Yu-Jie Chen, Rury. R. Holman

**Affiliations:** 1 Department of Endocrinology, Beijing Tongren Hospital, Capital Medical University, No. 1 Dongjiaomin Xiang, Dongcheng District, Beijing, 100730 China; 2 Department of Medical Records and Statistics, Beijing Ditan Hospital, Capital Medical University, Beijing, China; 3 Jinsong Community Health Service Center, Beijing, China; 4 Xinjiekou Community Health Service Center, Beijing, China; 5 Cuigezhuang Community Health Service Center, Beijing, China; 6 Yuetan Community Health Service Center of Fuxing Hospital, Capital Medical University, Beijing, China; 7 Aerospace Central Hospital, Beijing, China; 8 Sanlitun Community Health Service Center, Beijing, China; 9 Department of Endocrinology, Beijing Aerospace General Hospital, Beijing, China; 10 Jiangtai Community Health Service Center, Beijing, China; 11 Majiapu Community Health Service Center, Beijing, China; 12 Zuojiazhuang Community Health Service Center, Beijing, China; 13 Balizhuang Community Health Service Center, Beijing, China; 14 The First People’s Hospital of Dongcheng District, Beijing, China; 15 Dongfeng Community Health Service Center, Beijing, China; 16 Sijiqing Community Health Service Center, Beijing, China; 17 Diabetes Trials Unit, Radcliffe Department of Medicine, University of Oxford, Oxford, UK

**Keywords:** Cardiology, Endocrinology, Risk factors

## Abstract

Obesity increases the risk of developing cardiovascular disease and other metabolic diseases. We intended to compare three different anthropometric indicators of obesity, in predicting the incidence of cardiovascular events in Chinese type 2 diabetes. Beijing Community Diabetes Study was a prospective multi-center study conducted in Beijing community health centers. Type 2 diabetes patients from fourteen community health centers were enrolled at baseline. The primary endpoint was cardiovascular events. The upper quartile of neck circumference (NC) was set as greater NC. A total of 3299 diabetes patients were enrolled. In which, 941 (28.52%) had cardiovascular disease at baseline. Logistic analysis showed that central obesity (waist circumference (WC) above 90 cm in men and 85 cm in women) and greater NC were all related to baseline cardiovascular disease (adjusted OR = 1.49, and 1.55). After 10-year follow-up, 340 (10.31%) had cardiovascular events. Compared with patients without cardiovascular events, those having cardiovascular events had higher BMI, larger WC and NC. Cox regression analysis showed that greater WC and NC were all associated with the occurrence of cardiovascular events (adjusted HR = 1.41, and 1.38). A higher NC and WC might increase the risk of cardiovascular events by about 40% in type 2 diabetes patients in Beijing communities.

## Introduction

Obesity is a major public health problem worldwide^1^. Multiple studies have shown the association of obesity with increased risk of developing type 2 diabetes mellitus (T2DM), coronary heart disease, stroke and other metabolic syndrome^2–5^. Obesity has been evaluated using several anthropometric indexes, such as body mass Index (BMI), waist circumference (WC), and neck circumference (NC). NC, as an index for upper-body subcutaneous fat distribution, was reported by Sjostrom et al. in 1995^6^, and independently associated with cardiovascular risk factors in obese individuals. There were several studies to explore the association between NC and cardiovascular disease (CVD)^7–9^. In the Framingham heart study, it was found that NC was associated with CVD risk factors, however, not with the incidence of CVD events^7^. In a prospective cohort study, a greater NC was associated with a higher incidence of CVD events and mortality in high-risk population^8^. A cross-sectional study conducted on people with stable angina showed that NC was better in predicting the risk of coronary artery disease than BMI^9^. A report from the Beijing Community Diabetes Study (BCDS) baseline data found that NC was positively associated with central obesity and overweight in Chinese T2DM people^10^. The analysis from the BCDS 8-year follow-up data showed that NC was related to the incidence of CVD events following an 8-year management in T2DM^11^. CVD is one of macrovascular complications of T2DM and the leading cause of mortality in T2DM in China^12^. Several studies have reported the association of obesity indexes with CVD risk factors and CVD^11,13,14^. However, there is lack of prospective studies comparing the effects of BMI, WC, and NC on predicting the future CVD events in the Chinese T2DM patients. The aim of the present study was to evaluate the association between NC, WC, BMI and the occurrence of CVD events in T2DM following a 10-year management in Beijing communities.

## Results

### Baseline demographic characteristics

At baseline, a total of 3299 participants with T2DM were enrolled, in which, 1316 (39.89%) were males and 1983 (60.11%) were females. The mean age was 62.40 ± 10.48 years. The mean NC was 36.49 ± 3.73 cm. Higher NC (NC above the upper quartile) was observed among 671 (20.34%) participants, 670 (20.31%) were obese and 1978 (59.96%) had central obesity (WC ≥ 90 cm in men and ≥ 85 cm in women).

A total of 941 (28.52%) had a history of CVD at baseline. On comparing two BMI groups (BMI < 28 kg/m^2^ and BMI ≥ 28 kg/m^2^) with the prevalence of CVD, no statistically significant difference was observed (27.92% vs 30.90%, *P* = 0.128). With WC, participants with central obesity showed higher prevalence of CVD compared with those without central obesity (31.09 vs 24.68%, *P* < 0.001) and the association was found to be statistically significant. Also, participants with higher NC (NC above the upper quartile) showed statistically significant difference with the prevalence of CVD compared with those without high NC (27.17% vs 33.83%, *P* = 0.001).

Logistic analysis showed participants with central obesity and high NC were all associated with the baseline CVD (Crude OR = 1.38 (95% CI 1.18–1.61), 1.37 (95% CI 1.14–1.64), respectively, all *P* < 0.01). After adjusting age, diabetic duration, HbA1c, LDL, hypertension, gender, smoking, serum creatinine and aspirin treatment, these associations persisted (Adjusted OR = 1.49 (95% CI 1.12–1.97), 1.55 (95% CI 1.14–2.10), all *P* < 0.01). Obesity (BMI ≥ 28 kg/m^2^) was also associated with the baseline CVD (Adjusted OR = 1.47 (95% CI 1.08–2.00), *P* = 0.014).

### The occurrence of CVD events after 10-year follow-up

After 10-year follow-up, 340 participants experienced CVD events and the total incidence of CVD events was 10.31%. In men, the incidence of CVD events was 12.61%, whereas, among women it was 8.77% (*P* < 0.001). The total incidence of coronary events and cerebral events was 6.24% and 4.06% respectively.

Compare with participants without CVD events, people with CVD events were older and had greater BMI, WC and NC. However, no statistically significant difference was observed in NC between participants with and without CVD events. A statistically significant difference in SBP and HbA1c was observed (P < 0.05). People with CVD events had higher prevalence of baseline CVD (Table 1).

**Table 1:** Baseline characteristics in people with and without cardiovascular events.

	Total (n = 3299)	Without CVD group (n = 2959)	CVD group (n = 340)	t value	*P* value
Age (year)	62.40 ± 10.48	62.05 ± 10.63	65.40 ± 8.48	− 6.69	< 0.001
Men (n, %)	1316 (39.89)	1150 (38.86)	166 (48.82)	12.61^#^ (x^2^)	< 0.001
Women (n, %)	1983 (60.11)	1809 (61.14)	174 (51.18)		
Diabetic duration (year)	11.80 ± 98.31	12.16 ± 103.46	8.43 ± 9.77	1.73	0.083
BMI (kg/m^2^)	25.37 ± 3.47	25.32 ± 3.45	25.82 ± 3.56	− 2.49	0.013
WC (cm)	89.23 ± 9.43	89.00 ± 9.38	91.22 ± 9.69	− 4.13	< 0.001
NC (cm)	36.49 ± 3.73	36.45 ± 3.69	36.85 ± 4.02	− 1.77	0.077
SBP (mmHg)	129.36 ± 14.81	128.95 ± 14.61	132.96 ± 16.01	− 4.38	< 0.001
DBP (mmHg)	77.89 ± 8.96	77.92 ± 8.88	77.64 ± 9.64	0.50	0.615
FPG (mmol/L)	7.95 ± 2.65	7.94 ± 2.67	7.95 ± 2.42	− 0.04	0.967
PPG (mmol/L)	10.70 ± 5.01	10.66 ± 5.09	11.09 ± 4.26	− 1.67	0.095
HbA1c (%)	7.36 ± 1.64	7.34 ± 1.65	7.57 ± 1.56	− 2.44	0.015
TG (mmol/L)	1.84 ± 1.31	1.83 ± 1.31	1.90 ± 1.36	− 0.89	0.371
TC (mmol/L)	5.23 ± 1.24	5.22 ± 1.24	5.35 ± 1.23	− 1.81	0.070
LDL (mmol/L)	3.04 ± 0.94	3.03 ± 0.94	3.10 ± 0.88	− 1.18	0.237
HDL (mmol/L)	1.34 ± 0.47	1.35 ± 0.45	1.31 ± 0.61	0.94	0.350
Smoking (n, %)	543 (16.46)	487 (16.46)	56 (16.47)	0.00^#^ (x^2^)	0.995
Baseline CVD diseases (n, %)	941 (28.52)	785 (26.53)	156 (45.88)	56.02^#^ (x^2^)	< 0.001
Aspirin Treatment (n, %)	1694 (51.35)	1470 (49.68)	224 (65.88)	32.05^#^ (x^2^)	< 0.001
Lipid lowering treatment (n, %)	2185 (66.23)	1959 (66.20)	226 (66.47)	0.01^#^ (x^2^)	0.928

*CVD* cardiovascular disease, *BMI* body mass index, *WC* waist circumference, *NC* neck circumference, *SBP* systolic blood pressure, *DBP* diastolic blood pressure, *FPG*, fasting plasma glucose, *HbA1c* hemoglobin A1c, *TG* triglyceride, *TC* total cholesterol, *HDL* high-density lipoprotein cholesterol, *LDL* low-density lipoprotein cholesterol.

^#^Chi-square was used.

### Association of BMI, WC and NC with the occurrence of CVD events

**Figure 1:**
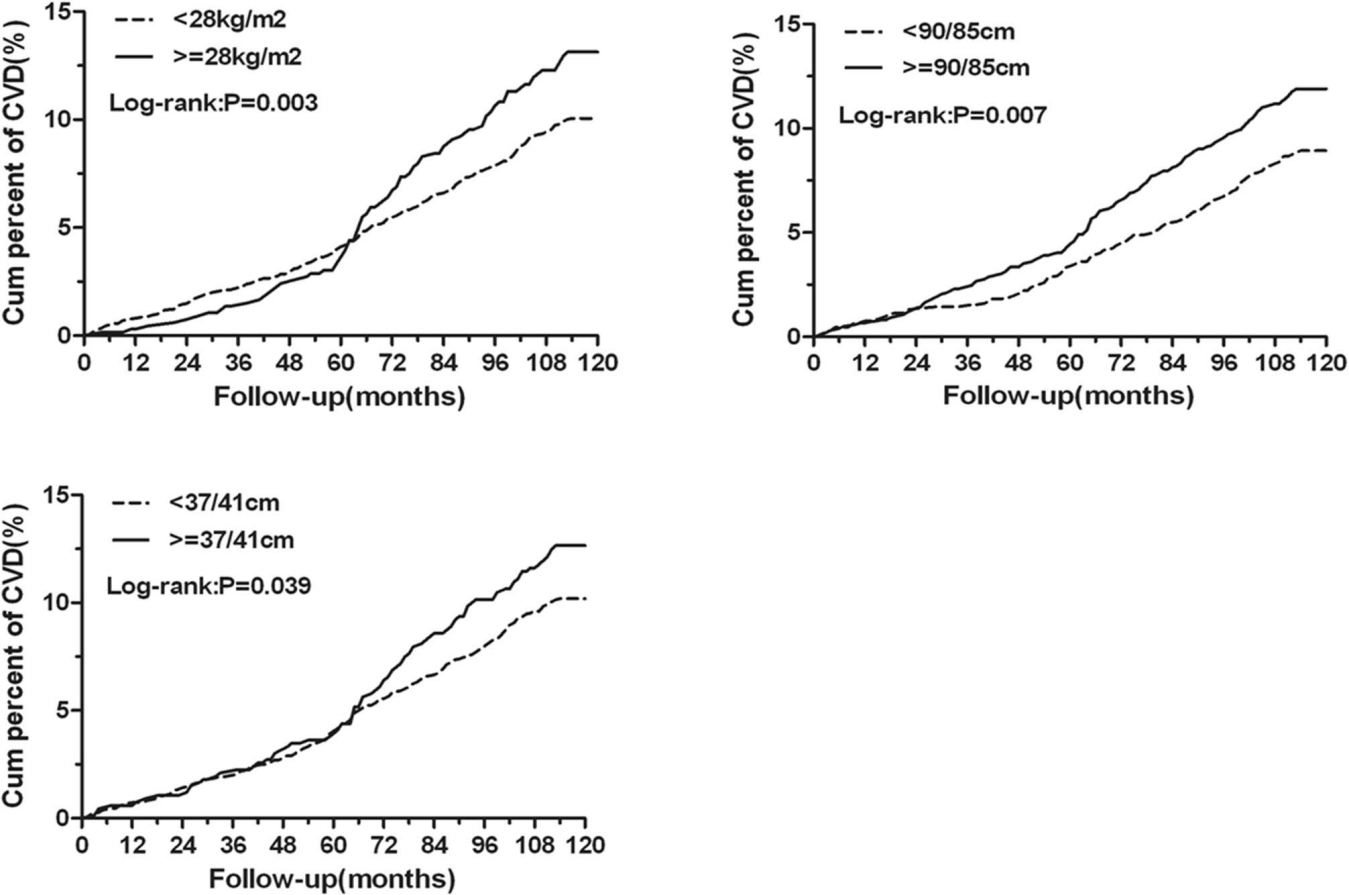
Kaplan–Meier estimates for body mass index, waist circumference, neck circumference with cardiovascular events during 10-year management. *CVD* cardiovascular diseases. This figure was drawn by using SAS for Windows version 9.2

Kaplan–Meier analysis showed that obesity, central obesity and high NC were associated with the cumulative incidence of CVD events, log-rank test confirmed these associations (*P* < 0.05, Fig. 1).

Cox regression analysis showed that central obesity and high NC were associated with the occurrence of CVD events after adjusting age, diabetic duration, HbA1c, LDL and hypertension (Adjusted HR = 1.39 (95% CI 1.07–1.82), 1.42 (95% CI 1.08–1.88), *P* < 0.05). Further adjusting baseline CVD, gender, smoking, serum creatinine and aspirin treatment, these associations persisted (Adjusted HR = 1.41 (95% CI 1.08–1.84), 1.38 (95% CI 1.04–1.83), *P* < 0.05). However, obesity was not associated with the occurrence of CVD events when obesity was diagnosed by measuring participant’s BMI (Adjusted HR = 1.31 (95% CI 0.98–1.75)).

## Discussion

In this study, the cumulative prevalence of CVD events increased with the BMI, WC and NC enlargement. Higher WC and NC were associated not only with the baseline CVD, but also with the incidence of CVD events during the 10-year management. Our study results showed that higher NC and WC might increase the risk of CVD and CVD events by about 40% in T2DM patients in Beijing communities. However, no association was found between higher BMI and the incidence of CVD events.

NC, a new indicator of upper body subcutaneous fat, is a reliable tool to screen obesity^15^. And NC was reported to be associated positively with central obesity and overweight in diabetes population^10,16^. Studies have shown positive correlation of NC measurement with multiple CVD risk factors, such as lipid profile, blood pressure and insulin resistance^7,17–19^. NC was also found to be related to metabolic syndrome in diabetes^20^. The relationship between NC and CVD was explored in different population^9,13,21^. A cross-sectional study showed that NC was associated with coronary artery disease in people with stable angina^9^. Another cross-sectional study in diabetes people found that NC was associated with the prevalence of CVD^21^. A meta-analysis including eight observation studies found that NC was related to CVD, especially when CVD was diagnosed by coronary angiography^13^. There were prospective studies to explore the association between NC and CVD events^8,11^. In a prospective cohort study involving high-risk CVD patients, higher NC was related to increased incidence of future fatal and non-fatal CVD events^8^. NC was also found to be associated with the incidence of CVD events in T2DM^11^.

WC has been used to measure abdominal adiposity and it is one of the indicators of obesity in clinical practice as recommended by the Chinese guideline for type 2 diabetes^22^. WC was associated with CVD risk factors^23,24^. A study conducted by Yang Liu et al. among the Chinese population suggested that WC was the predictor of multiple metabolic risk factors such as blood pressure, glucose, and triglyceride^23^. WC was found to be associated with blood pressure, glucose and total cholesterol in Vietnam subjects^24^. WC was reported to be related to the prevalence of CVD in an international study conducted in primary care^25^. In a prospective study conducted in Japanese diabetes people, WC was not a predictive indicator for future CVD events despite WC was associated with CVD risk factors^26^. However, In ADVANCE (Action in Diabetes and Vascular disease: preterAx and diamicroN-MR Controlled Evaluation) trial, an international cohort study, WC was related to CVD events in subjects with T2DM^27^.A meta-regression analysis showed WC was positively associated with the risk of CVD events, i.e. for 1 cm increase in WC, there would be 2% increase in the relative risk of CVD events^28^.

Though BMI was widely used in clinical practice to measure obesity, BMI could not reflect the body fat distribution. Also, BMI has been sub-optimally correlated with obesity in CVD patients due to variation in body fat distribution and metabolic risk factors associated^29,30^. Meta-analysis showed that increase in BMI was associated with the risk of CVD events^31–33^. However, Previous studies have found that high BMI was associated with lower mortality among patients with CVD and in other population as well^29^.

There were studies comparing the effects of different indicators of obesity on CVD^25,34^. An international cross-sectional study showed that WC had a stronger relationship with CVD compared with BMI^25^. A study conducted by Canoy et al.^34^ assessing distribution of body fat and risk of CVD found that abdominal obesity was the stronger predictor of CVD compared with BMI. Evaluation of different anthropometric indicators to assess CVD events among diabetes patients is limited. A prospective study conducted by Cho et al.^35^ showed a strong association of BMI with increased risk of CVD events in diabetes women with no history of CVD. However, in ADVANCE trial, WC, not BMI was related to CVD events in subjects with T2DM^27^. This finding is in line with our study result. In our study, WC, not BMI was associated with the increased risk of CVD events in T2DM people. There is lack of prospective cohort studies in T2DM comparing NC, WC and BMI on predicting the future CVD events. Our study reported the effects of BMI, WC, and NC on the incidence of CVD events in T2DM. In our study, NC and WC were found to have comparable association with the occurrence of CVD events in Chinese T2DM. The measurement of WC is not always practical in clinical practice, especially in winter. And it may differ between and after meal, especially in obese people. Our results indicated that NC might be used as a simple test for identifying obesity in predicting CVD events in future clinical practice, especially when the measurement of WC was inconvenient.

This study had some limitations. Initially, all the people with T2DM were managed by general practitioners according to the Chinese guideline for type 2 diabetes. The antiglycemic, antihypertensive and statin medication might have changed during the 10-year follow-up to result in HbA1c, blood pressure and LDL levels under control according to the Chinese guideline for type 2 diabetes. This might affect the incidence of CVD events compared with T2DM patients without management. Secondly, T2DM itself is a risk factor for CVD events^36^. T2DM people had higher prevalence of CVD. In our study, 28.52% people had CVD when enrollment, which may increase the risk of CVD events. Thirdly, about 16% of the patients in our study did not participate in the last visit in 2018, which may affect the incidence of CVD events reported in this study. The incidence of CVD events in this study may be underestimated.

In conclusion, in this prospective multi-center study, during the 10-year follow-up, greater NC and WC were associated not only with the prevalence of CVD, but also with the occurrence of CVD events in Chinese T2DM population. Moreover, NC and WC had comparable association with the occurrence of CVD events in Chinese T2DM. Greater NC and WC might increase the risk of CVD events by about 40%. Further large-scale prospective studies are needed to confirm this relationship.

## Methods

This prospective, multi-center study (Beijing Community Diabetes Study, BCDS) was conducted at fourteen community health centers such as, Cuigezhuang, Jinsong, Xinjiekou, Yuetan, Donggaodi, Yongdinglu, Sanlitun, Jiangtai, Shazikou, Balizhuang, Zuojiazhuang, Dongfeng, Sijiqing and Majiapu community health center associated with Beijing Tongren Hospital. Multistage random sampling method was used to recruit T2DM patients at baseline in 2008 and the patients were followed up for next 10 years. T2DM patients aged 20 to 80 years and those who residing in the same community for at least 5 years were included in the study. Patient with hepatic failure, renal failure, severe disabilities, schizophrenia or goiter were excluded. Details of study design, methods and population of BCDS had been reported elsewhere^10,37^. A total of 140 participants with incomplete baseline data such as height, weight, neck circumference, waist circumference and lab values were excluded. There were 3299 DM participants were included in the analysis and 84% participants participated in the last visit in 2018.

The ethical approval was obtained from the Ethics Committee of Beijing Tongren Hospital, Capital Medical University. The study was conducted in accordance with the provisions of the Declaration of Helsinki and its subsequent revisions. Written informed consent was obtained from all the participants.

A physical examination including anthropometric parameters like BMI, WC and NC was performed according to the study protocol at baseline and subsequent follow-up visits. BMI was calculated by dividing body weight (kg) with the height squared (m^2^) along with light clothing and without shoes. WC (cm) was measured horizontally between the lower rib margin and the iliac crest in the mid-axillary line, with the subject in standing position and fasting. NC (cm) was measured horizontally at the upper margin of the laryngeal prominence (Adam’s apple) with people standing, head erecting and eye facing forward according to the protocol^10,37^. Patients with BMI ≥ 28 kg/m^2^ was diagnosed obese and WC ≥ 90 cm in men and 85 cm in women was set as the criteria of diagnosing central obesity according to the *Chinese guidelines for Type 2 Diabetes*^22^. NC above the upper quartile was defined as high NC. CVD was defined if the patients had been diagnosed in a top tier hospital having coronary heart disease, myocardial infarction, angina pectoris, stroke, or other clinical manifestations of CVD.

Over the follow-up period of 10 years, all the T2DM patients were seen by a collaborative team at least four times each year^32^. The study outcome was the occurrence of CVD events including coronary events (cardiac death, myocardial infarction, unstable angina pectoris, hospitalization for heart failure and coronary revascularization) and cerebral events (stroke, transient ischemic attack). Stroke was confirmed by cranial Computed Tomography or Magnetic Resonance Imaging.

### Statistical analysis

The database was established using Epi-Data software (version 3.0). SAS software (SAS Institute Inc., Cary, NC) was used in statistical analysis. All the T2DM patients were categorized into the CVD group and without CVD group. An unpaired student *t*-test and chi-square test was used to compare the continuous and categorical data respectively at baseline and follow-up. The association between BMI, WC, NC and baseline CVD was assessed using Logistic regression to estimate the odds ratio (OR) and 95% confidence interval (CI). Kaplan–Meier analysis and log-rank test were used to assess the associations between BMI, WC, NC and the cumulative percentage of CVD events. The proportional hazards assumption of the Cox model was tested (Supplementary Table S1, Fig. S1). The proportional hazards Cox regression analysis was used to estimate the effects of BMI, WC and NC on CVD events risk and hazard ratio (HR) with 95% CI was reported. Last Observation Carry Forward (LOCF) was used for missing data during the 10-year follow-up. The physical and lab examination values were imputed by using the data of the most recent previous visit. The study outcome was imputed by the most recent reported outcome. The time of dropout was the time of the most recent previous visit. A *P* value < 0.05 in 2-tailed tests was considered to be statistically significant.

## Supplementary Information

Supplementary Information.
