# Supplementary material for: Neck circumference and waist circumference associated with cardiovascular events in type 2 diabetes (Beijing Community Diabetes Study 23)
Source: Sci Rep. 2021 May 4;11:9491. doi: 10.1038/s41598-021-88927-9 (PMC8097072; doi:10.1038/s41598-021-88927-9)
Supplement: Supplementary file 1 — Supplementary Information. [file 41598_2021_88927_MOESM1_ESM.docx]

Neck circumference and waist circumference associated with cardiovascular events in type 2 diabetes (Beijing Community Diabetes Study 23)

Guang-Ran Yang^1*^, Ming-Xia Yuan^1^, Gang Wan^2^, Xue-Lian Zhang^1^, Han-Jing Fu^1^, Shen-Yuan Yuan^1*^, Liang-Xiang Zhu^1^, Rong-Rong Xie^1^, Jian-Dong Zhang^3^, Yu-Ling Li^4^, Yan-Hua Sun^5^, Qin-Fang Dai^6^, Da-Yong Gao^7^, Xue-Li Cui^8^, Jian-Qin Gao^9^, Zi-Ming Wang^10^, Ying-Jun Chen^11^, Yong-Jin Li^3^, Dong-Ming Hu^12^, Juan Gao^13^, Ying Gao^14^, Jie Miao^15^, Yu-Jie Chen^16^, Rury. R. Holman^17*^

^1^ Department of Endocrinology, Beijing Tongren Hospital, Capital Medical University, Beijing, China

^2^ Department of Medical Records and Statistics, Beijing Ditan Hospital, Capital Medical University, Beijing, China

^3^ Jinsong Community Health Service Center, Beijing, China

^4^ Xinjiekou Community Health Service Center, Beijing, China

^5^ Cuigezhuang Community Health Service Center, Beijing, China

^6^ Yuetan Community Health Service Center of Fuxing Hospital, Capital Medical University, Beijing, China

^7^ Aerospace Central Hospital, Beijing, China

^8^ Sanlitun Community Health Service Center, Beijing, China

^9^ Department of Endocrinology, Beijing Aerospace General Hospital, Beijing, China

^10^ Jiangtai Community Health Service Center, Beijing, China

^11^ Majiapu Community Health Service Center, Beijing, China

^12^ Zuojiazhuang Community Health Service Center, Beijing, China

^13^ Balizhuang Community Health Service Center, Beijing, China

^14^ The First People's Hospital of Dongcheng District, Beijing, China

^15^ Dongfeng Community Health Service Center, Beijing, China

^16^ Sijiqing Community Health Service Center, Beijing, China

^17^ Diabetes Trials Unit, Radcliffe Department of Medicine, University of Oxford, UK

| **Supplementary Table S1:** The results of time-dependent covariable for neck circumference and time in the Cox model | | | | | | | |
| --- | --- | --- | --- | --- | --- | --- | --- |
|  | B | SE | Wald | P | HR | 95%CI for HR | |
|  |  |  |  |  |  | Lower | Upper |
| Time_COV*Neck circumference | 0.00 | 0.00 | 0.61 | 0.436 | 1.00 | 1.00 | 1.01 |
| Neck circumference | 0.00 | 0.31 | 0.00 | 0.994 | 1.00 | 0.54 | 1.85 |

**
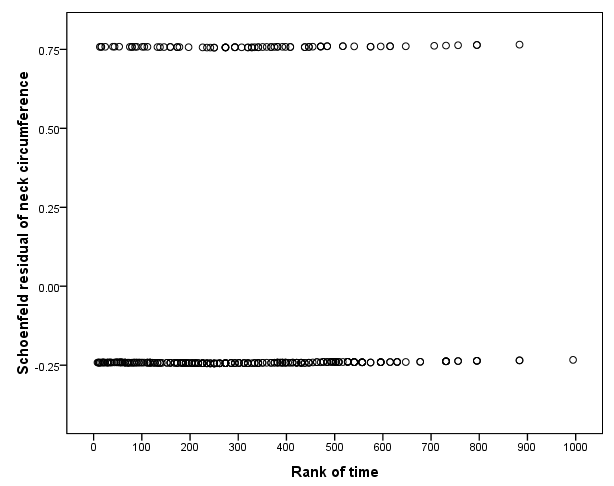
**

**Supplementary Figure S1:** Plots of Schoenfeld residual of neck circumference versus rank order of time

There was no statistically significant correlation between Schoenfeld residual of neck circumference and the rank order of time (r=0.04，P=0.42).
